# Effects of cooking with solid fuel on hearing loss in Chinese adults—Based on two cohort studies

**DOI:** 10.1038/s41598-024-61498-1

**Published:** 2024-05-10

**Authors:** Xue-yun Mao, Miao Zheng, Jun-ping Wang, Shou Kou, Wei-hao Wang, Jun-jie Lin, Ru-cheng Chen, Qing-hua Sun, Wei-jun Zheng

**Affiliations:** Zhejiang Chinese Medicine University, Hangzhou, China

**Keywords:** Hearing loss, Solid fuel, Cohort study, CHARLS, CLHLS, Environmental impact, Epidemiology

## Abstract

The association between cooking fuel and hearing loss still needs more research to clarify, and two longitudinal cohort studies were explored to find if solid fuel use for cooking affected hearing in Chinese adults. The data from Chinese Health and Retirement Longitudinal Survey (CHARLS) and Chinese Longitudinal Healthy Longevity Survey (CLHLS) were analyzed. Participants (older than 18) without hearing loss at baseline and follow-up visits were included, which were divided into clean fuel and solid fuel groups. Hearing loss rate was from follow-up visits (both in year 2011) until the recent one (year 2018 in CHARLS and 2019 in CLHLS). Cox regressions were applied to examine the associations with adjustment for potential confounders. Fixed-effect meta-analysis was used to pool the results. A total of 9049 participants (average age 8.34 ± 9.12 [mean ± SD] years; 4247 [46.93%] males) were included in CHARLS cohort study and 2265 participants (average age, 78.75 ± 9.23 [mean ± SD] years; 1148 [49.32%] males) in CLHLS cohort study. There were 1518 (16.78%) participants in CHARLS cohort and 451 (19.91%) participants in CLHLS cohort who developed hearing loss. The group of using solid fuel for cooking had a higher risk of hearing loss (CHARLS: HR, 1.16; 95% CI 1.03–1.30; CLHLS: HR, 1.43; 95% CI 1.11–1.84) compared with the one of using clean fuel. Pooled hazard ratio showed the incidence of hearing loss in the solid fuel users was 1.17 (1.03, 1.29) times higher than that of clean fuel users. Hearing loss was associated with solid fuel use and older people were at higher risk. It is advised to replace solid fuel by clean fuel that may promote health equity.

## Introduction

Hearing loss is the fourth leading cause of disability worldwide^1^ and the second most common nonfatal disorder that affects Chinese population. Currently, the World Health Organization (WHO) reported^2^ that 430 million of them had moderate or severe hearing loss in their better-hearing ears. In China, about 11 percent of adults suffered from disabling hearing loss according to the Second Sample Survey on Disability in 2006^3^. As an age-related disease, hearing loss affects approximately one-third of the adults from 61 to 70 years old and more than 80 percent of those older than 85 years. It is reported that the adults with hearing loss are characterized by low education and lower-class occupation^4^. Besides, the unemployment rate for those with hearing loss is higher than for their normal hearing counterparts in both developed and developing countries^5^. Compared with the adults of normal hearing, those with hearing loss have a higher risk of psychiatric disorders, such as isolation, depression and anxiety^6^. Furthermore, a prospective community-based study investigated 3777 participants aged 65 and older and found an increased risk of disability and dementia in those participants with hearing problems^7^, which may increase the risk of death. Nowadays, the cause of sudden hearing loss is not known but is presumed to be viral, vascular or autoimmune, and the main causes of sensorineural hearing loss are degenerative processes associated with aging, genetic mutations, noise exposure, exposure to therapeutic drugs that have ototoxic side effects, and chronic conditions^8^. People are often unaware of minor hearing loss and they may seek for help only when there is obvious hearing loss.

Solid fuel, an important source of severe Household Air Pollution (HAP) that is linked to many adverse health outcomes, has been widely consumed around the world^9^. Solid fuel combustion produces mixture, such as fine particulate matter (PM_2.5_) and carbon monoxide, which are also the main sources of polycyclic aromatic hydrocarbons (PAHs) in developing countries^10^. Accumulated indoor, may these pollutants and their joint effect that raise the risk of hearing loss. Some experiments and epidemiology studies found the potential links between single combustion component and hearing loss. A cohort study enrolled 8835 adults showed that the use of solid fuel for cooking and heating is closely associated with poor hearing function^11^. Solid fuel may impact the hearing through other diseases, epidemiological studies have shown that using solid fuels for cooking increases the mortality of cardiovascular^12,13^ and respiratory diseases^14^, especially in women, children, and the elderly. In addition, using solid fuel indoor could cause cognitive impairment or cognitive decline in the elderly^15^. Nowadays, approximately 2.5 billion individuals globally use solid fuel for cooking^16^ and it is widely used in China because of its affordability and availability, especially in rural areas^17^. Thus, more evidences are needed to evaluate the association between solid fuels and hearing loss especially at the crowd level. Based on two large Chinese databases (CHARLS and CLHLS), this study aimed to clarify the association between the use of solid fuels for cooking and the incidence of hearing loss in Chinese adults.

## Materials and methods

### Study population

The study population was derived from the China Health and Retirement Longitudinal Study (CHARLS) and the Chinese Longitudinal Healthy Longevity Survey (CLHLS). Both of them are publicly available, which were granted to be accessed by the application. The data from CLHLS survey already obtained the ethical approval and informed consent, and was approved by research ethics committees of Duke University and Peking University (IRB00001052-13074). The CHARLS study was approved by the Biomedical Ethics Review Board of Peking University (IRB00001052-11015). And each participant signed an informed consent form.

CHARLS was a large-scale cohort study that investigated about 18,000 individuals mostly aged 45 and above covers 150 counties, 450 communities (villages) in 28 provinces (including autonomous regions and municipalities) of China, which was the first national survey that was conducted initially in 2011, then every 2–3 years. Follow-up surveys have been conducted in 2013, 2015, and 2018 by well-trained interviewers at participants’ designated locations via structured questionnaires. CLHLS is a large-scale survey in elderly population mostly 65 and older that was conducted in 23 provinces, municipalities and autonomous regions in China. It was started in 1998, then follow-up surveys were conducted in 2002, 2005, 2008–2009, 2011–2012, 2014, and 2017–2019 by trained and qualified interviewers at participants’ suggested locations via structured questionnaires. Persons who meet the age requirements are randomly selected from each locality and only one person per household is selected. Themselves or their family members answer the questionnaire.

To make sure the sample size effective, we used the formula below to calculate it. As the incidence rate of hearing loss is hard to get that the prevalence rate was used to estimate. According to the latest investigation around the four provinces about the prevalence of hearing disorders in China based on “WHO Ear and Hearing Disorders Survey Protocol”, the standard prevalence rate of hearing loss is 15.84%^18^. As for CHARLS, we assume RR = 1.2, *p*_*0*_ = 15.84%, the minimum sample size is 3744. As for CLHLS, we assume RR = 1.5, *p*_*0*_ = 15.84%, the minimum sample size is 659. Finally, we will make a test efficiency to verify the validity of sample size.

$$ n = (\frac{{2u_{{{1 - }\alpha /2}} \sqrt {\overline{p} \overline{q} } + u_{\beta } \sqrt {p_{0} q_{0} + p_{1} q_{1} } }}{{\left[ {p_{1} - p_{0} } \right]}})^{2} ,\,\alpha = {0}{\text{.05}},\,\beta = 0.1 $$

This study analyzed the two cohorts over the same time period (2011–2019). At baseline (2011–2012), a total of 17,705 participants were investigated in CHARLS cohort and 9765 participants in CLHLS cohort. Then, follow-up visit surveys were selected (CHARLS in 2013, 2015, and 2018 and CLHLS in 2014–2015, 2017–2019) to build fixed queue. Inclusion criteria is adults with normal hearing at baseline. At first, people without the data of fuel exposure, hearing data and using the hearing aid needed to be excluded, the remainder included in the cross-sectional analysis. And then, by inclusion criteria, a total of 9049 participants in CHARLS cohort and 2265 participants in CLHLS cohort were enrolled in the current analysis.

**Figure 1:**
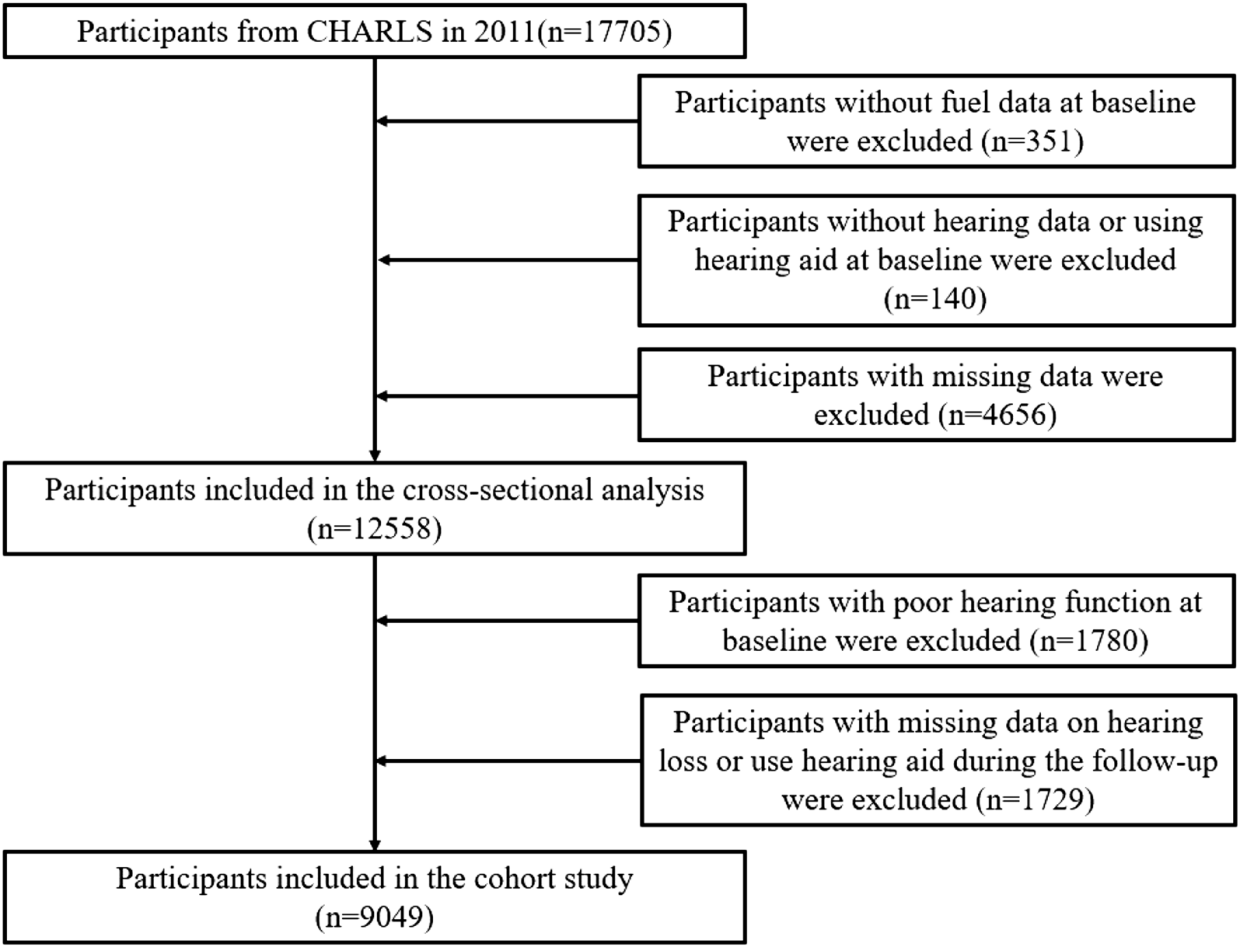
CHARLS flowchart.

**Figure 2:**
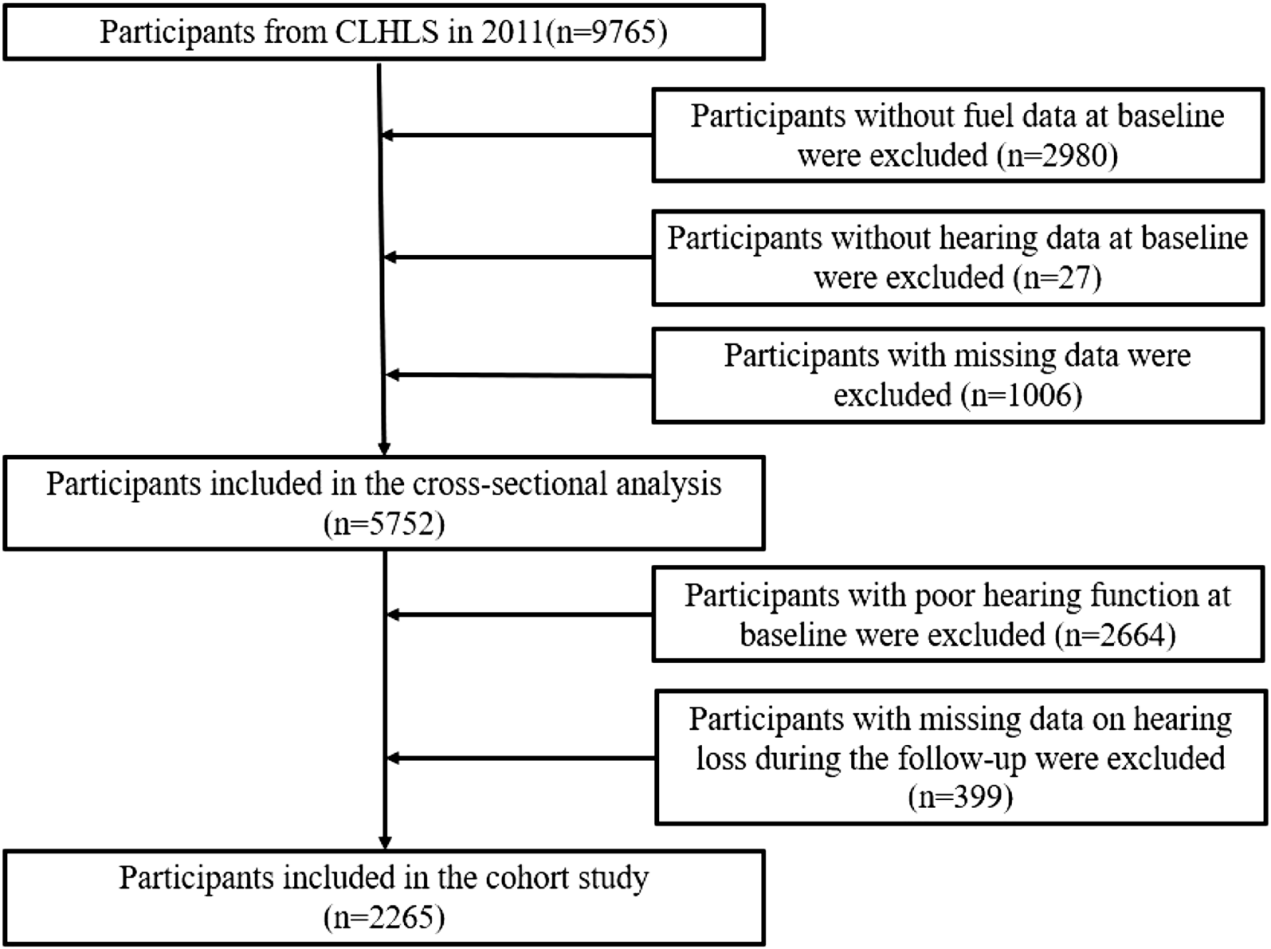
CLHLS flowchart.

More details are shown in Figs. 1 and 2.

### Assessment of cooking fuel and outcome

Energy source for cooking was assessed through questions of “Which fuels are normally used for cooking in your home?” in CLHLS 2011 questionnaire^19^ and “What is the main source of cooking fuel?” in CHARLS 2011 questionnaire^20^. Participants were divided into two categories by the sources of cooking energy: solid fuel (firewood and coal) and clean fuel (piped natural gas, electricity and solar energy), because clean fuel tends to generate much less air pollutants than solid fuel.

Participants who answered “Yes” of the question “Have any difficulty with his/her hearing” in CLHLS were assessed as hearing loss. CHARLS classified hearing loss as “very poor” when participants were questioned “Is your hearing very good, good, fair, poor, or very poor”. The onset of hearing loss was referred to the first occurrence of hearing loss during the follow-up.

### Statistical analysis

CHARLS and CLHLS cohort studies were analyzed separately. Baseline characteristics of the study population were described as means (SDs) or percentages by categorical cooking fuel exposure. T-test or χ2 test was used to compare the difference between the people using solid fuel or clean fuel for cooking. Log-Rank was used to analyze relativity between cooking fuel and hearing function. And then we calculated the power of two cohort study. Cox proportional hazards models were used to estimate hazard ratios (HRs) and 95% CI for the associations of fuel use and hearing dysfunction. The variables of CLHLS and CHARLS cohort studies were defined and classified consistently. Hearing loss is unavoidable with aging and women are the ones who cook the most in Chinese family, based on the first model (only cooking fuel), the second cox proportional hazards model adjusted age and gender separately was necessary. Multivariate models were stratified by gender (male/female), age, marital status (married, single), educational level (below primary school, primary school and above), smoking (never, quit, smoking), alcohol consumption (yes, no), and body mass index (BMI). In addition, hypertension (yes, no) and diabetes (yes, no) were adjusted based on model-3 because hypertension and diabetes were not definitive mediators. The hazard ratios (HRs) of different models were separately analyzed in CHARLS and CLHLS cohorts, and then pooled the cohort-specific HRs by fixed effect models to obtain a summarized risk estimate. All *P* values were 2-sided and *P* < 0.05 denoted statistical significance. Analyses were performed using SPSS (IBM SPSS Statistics 26) and PASS (PASS 11), graphs were plotted using R version 4.0.5 (R Foundation).

## Results

### Participant demographic data

A total of 9049 participants were included in CHARLS cohort study, with an average age of 58.34 ± 9.12 years, and 4247 (46.93%) males. At baseline, 5093 (56.28%) participants were exposed to solid fuels, and 3956 (43.72%) people were exposed to clean fuels. During the follow-up period from 2011 to 2018 (mean follow-up time: 6.48 ± 0.02 years, 95% CI 6.45–6.51 years), 1518 participants developed hearing loss, with a cumulative incidence rate of 16.78%. Baseline demographic characteristics according to household cooking fuels are shown in Table 1. Participants exposed to solid fuel were older than that of clean fuel users, and most of them lived in rural areas, single, below primary school education, never smoking, without hypertension and diabetes, and with lower BMI (*P* < 0.05).

**Table 1:** Baseline population characteristics based on cooking fuel.

	CHARLS				CLHLS			
	Clean fuel	Solid fuel	χ2/t	*P*	Clean fuel	Solid fuel	χ2/t	*P*
Gender n (%)			1.28	0.26			0.56	0.45
Male	1830(46.26)	2417(47.47)			340(47.49)	808(51.20)		
Female	2126(53.74)	2676(52.53)			347(52.51)	770(48.80)		
Age (x ± s)	57.42 ± 9.25	59.05 ± 8.95	8.39	< 0.01	79.05 ± 8.98	78.62 ± 9.30	− 1.01	0.31
Residence n (%)			1378.87	< 0.01			579.96	< 0.01
Rural	2517(63.62)	4813(94.50)			423(61.57)	1552(98.35)		
Urban	1439(26.38)	280(5.50)			264(38.43)	26(1.65)		
Marital status n (%)			0.21	0.65			5.12	0.02
Married	3505(88.60)	4528(88.91)			323(47.01)	661(41.89)		
Single	451(11.40)	565(11.09)			364(52.99)	917(58.11)		
Education level n (%)			353.91	< 0.01			56.71	< 0.01
Below primary school	1307(33.04)	2691(52.84)			267(11.79)	828(36.56)		
Primary school and above	2649(66.96)	2402(47.16)			420(18.54)	750(33.11)		
Live alone n (%)			0.01	0.92			6.24	0.01
Yes	3519(88.96)	4534(89.02)			107(15.57)	316(20.03)		
No	437(11.04)	559(10.98)			580(84.43)	1262(79.99)		
Smoking n (%)			18.62	< 0.01			13.50	< 0.01
Never smoking	2503(63.27)	3035(59.59)			430(62.59)	988(62.61)		
Quit smoking	332(8.39)	400(7.85)			126(18.34)	210(13.31)		
Smoking	1121(28.34)	1658(32.55)			131(19.07)	380(24.08)		
Drinking n (%)			0.49	0.48			1.01	0.31
No	2628(66.43)	3419(67.13)			559(81.37)	1255(79.53)		
Yes	1328(33.57)	1674(32.87)			128(18.63)	323(20.47)		
Hypertension n (%)			4.02	0.045			17.64	< 0.01
With	950(24.01)	1132(22.23)			240(34.93)	414(26.24)		
Without	3006(75.99)	3961(77.77)			447(65.07)	1164(73.76)		
Diabetes n (%)			13.26	< 0.01			21.58	< 0.01
With	255(6.45)	239(4.69)			50(7.29)	47(2.98)		
Without	3701(93.55)	4854(95.31)			637(92.71)	1531(97.02)		
BMI (x ± s)	24.15 ± 4.01	23.20 ± 3.86	− 11.35	< 0.01	22.4 ± 3.94	21.67 ± 4.04	− 4.17	< 0.01

A total of 2265 participants were included in CLHLS cohort study, with a mean age of 78.75 ± 9.23 years, and 1148 (49.32%) males. Among the participants, 1578 (69.67%) were exposed to solid fuels at baseline and 687 (30.33%) were exposed to clean fuels. During the follow-up period from 2011 to 2019 (mean follow-up time: 7.09 ± 0.04 years, 95% CI 7.01, 7.17), 451 people developed hearing loss, with a cumulative incidence rate of 19.91%. Baseline demographic characteristics based on household cooking fuel are shown in Table 1. The majority of solid fuel users lived in rural, single, below primary school education, not living alone, never smoking, without hypertension and diabetes, and with lower BMI (*P* < 0.05).

### The associations between hearing loss and baseline demographic characteristics

Log Rank test was used to analyze whether there was a difference between the incidence of hearing loss and baseline demographic characteristics, as shown in Table 2. For CHARLS cohort study, hearing loss occurred in 534 clean fuel users during the 8 years of follow-up, and its cumulative prevalence rate was 13.55%. A total of 982 participants had hearing loss among solid fuel users, its cumulative prevalence rate was 19.28%, which was higher than that in clean fuel users. Log-Rank test showed different types of cooking fuel were associated with hearing loss condition (*P* < 0.05). Besides, the elder, women, and people living in rural, married, below primary school education, living alone, drinking, with hypertension, and lower BMI (*P* < 0.05) account for the vast majority among people occurred from hearing loss.

**Table 2:** Baseline population characteristics based on hearing function.

	CHARLS				CLHLS			
	Normal hearing	Hearing loss	χ2/t	*P*	Normal hearing	Hearing loss	χ2/t	*P*
Fuel			49.43	< 0.01			13.36	< 0.01
solid fuel	4111(54.59)	982(64.69)			1239(68.30)	339(24.83)		
clean fuel	3420(45.41)	536(35.31)			575(31.70)	112(75.17)		
Gender n (%)			8.64	< 0.01			2.01	0.16
Male	3594(47.72)	653(43.02)			911(50.22)	237(52.55)		
Female	3937(52.28)	865(56.98)			903(49.78)	214(47.45)		
Age (x ± s)	57.94 ± 9.09	60.31 ± 9.02	191.99	< 0.01	78.73 ± 9.49	78.87 ± 8.07	74.19	0.05
Residence n (%)			55.29	< 0.01			3.91	0.05
Rural	5987(79.49)	1343(88.47)			1572(86.66)	403(89.36)		
Urban	1544(20.51)	175(11.52)			242(13.34)	48(10.64)		
Marital status n (%)			11.49	< 0.01			2.73	0.01
Married	6713(86.94)	1320(89.14)			1027(56.62)	254(56.32)		
Single	198(13.04)	818(10.86)			787(43.38)	197(43.68)		
Education level n (%)			131.1	< 0.01			3.87	0.05
Below primary school	3132(41.59)	866(57.05)			865(47.68)	230(52.32)		
Primary school and above	4399(58.41)	652(42.95)			949(52.32)	221(47.68)		
Live alone n (%)			13.01	< 0.01			1.39	0.24
Yes	6732(89.39)	1321(87.02)			331(18.25)	92(20.40)		
No	799(10.61)	197(12.98)			1483(81.75)	359(79.60)		
Smoking n (%)			2.17	0.34			0.51	0.78
Never smoking	4577(60.78)	961(63.31)			1142(62.95)	276(61.20)		
Quit smoking	616(8.18)	116(7.64)			269(14.83)	67(14.86)		
Smoking	2338(31.05)	441(29.05)			403(22.22)	108(23.95)		
Drinking n (%)			15.05	< 0.01			0.56	0.46
No	4967(65.95)	1080(71.15)			1463(80.65)	351(77.83)		
Yes	2564(34.05)	438(28.85)			351(19.35)	100(22.17)		
Hypertension n (%)			12.84	< 0.01			0.01	0.94
With	1686(22.39)	396(26.09)			520(28.67)	134(29.71)		
Without	5845(77.61)	1122(73.91)			1294(71.33)	317(70.29)		
Diabetes n (%)			0.033	0.86			1.07	0.30
With	412(5.47)	82(5.40)			79(4.36)	18(3.99)		
Without	7119 (94.53)	1436(94.60)			1735(95.64)	433(96.01)		
BMI (x ± s)	23.67 ± 3.95	23.32 ± 3.97	− 3.17	< 0.01	21.91 ± 4.05	21.87 ± 3.93	1072.48	< 0.01

For CLHLS cohort study, hearing loss occurred in 339 participants with solid fuel and 112 participants with clean fuel, the cumulative prevalence rate of hearing loss was 21.48% in solid fuel users and 16.30% in clean fuel users. Log-Rank test showed different types of cooking fuel were associated with hearing loss condition (*P* < 0.05). The characteristics of people with hearing loss were living in rural, married, below primary school education and lower BMI (*P* < 0.05).

### Power of two cohort study to explore the difference

**Figure 3:**
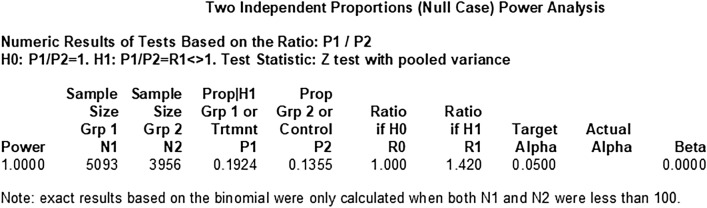
The power of CHARLS.

**Figure 4:**
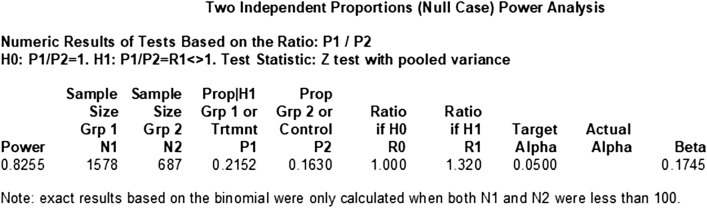
The power of CLHLS.

To clarify that the sample size has the ability to get a reliable result, we calculated the power of two cohort study. Both of them choose the *α* = 0.05. We get the result that using the sample size (n = 9049) in CHARLS to find the difference of hearing loss between the solid fuel and clean fuel is reliable, its power is 1. And in CLHLS, using the sample size (n = 2265) is not reliable as well as CHARLS, its power is 0.8255, but there is still some credibility. More details are shown in Figs. 3 and 4.

### Cooking fuel and hearing loss

The relationship between cooking fuel and hearing loss was shown in Table 3. In CHARLS, after multivariate confounding factors (sex, age, residence, marital status, education level, living status, smoking status, alcohol consumption, hypertension, diabetes, and BMI) were adjusted, fuel remained an independent contributor to hearing loss (*P* < 0.05). Compared with the participants exposed to clean fuel, those cooked using solid fuel had an increased risk of hearing loss by 16% (HR: 1.16, 95% CI 1.03–1.30, *P* < 0.05).

**Table 3:** Association analysis of fuel and hearing loss.

Database	Fuel	Model 1 HR (95% CI)^a^	Model 2 HR (95% CI)^b^	Model 3 HR (95% CI)^c^	Model 4 HR (95% CI)^d^
CLHLS	Clean fuel	Reference	Reference	Reference	Reference
	Solid fuel	1.45(1.17,1.80)	1.47(1.18,1.81)	1.43(1.11,1.83)	1.43(1.11,1.84)
CHARLS	Clean fuel	Reference	Reference	Reference	Reference
	Solid fuel	1.44(1.29,1.60)	1.38(1.24,1.53)	1.16(1.03,1.30)	1.16(1.03,1.30)

^a^Not adjusted.

^b^Adjust for gender and age.

^c^On the basis of Model 2, adjusted for urban and rural areas, marital status, education level, living alone, smoking, drinking and BMI classification.

^d^On the basis of Model 3, adjusted for hypertension and diabetes.

China Health and Retirement Longitudinal Survey (CHARLS) and Chinese Longitudinal Healthy Longevity Survey (CLHLS).

For CLHLS, the models built were similar to the ones in CHARLS, multivariate confounding factors (sex, age, residence, marital status, education level, living status, smoking status, alcohol consumption, hypertension, diabetes, and BMI) were adjusted in Model 4, and the results also showed that fuel made a difference to hearing loss (*P* < 0.05). The participants had a 43% increased risk of hearing loss compared to those exposed to clean fuels (HR: 1.43, 95% CI 1.11–1.84, *P* < 0.01).

### Merging of research results

**Figure 5:**
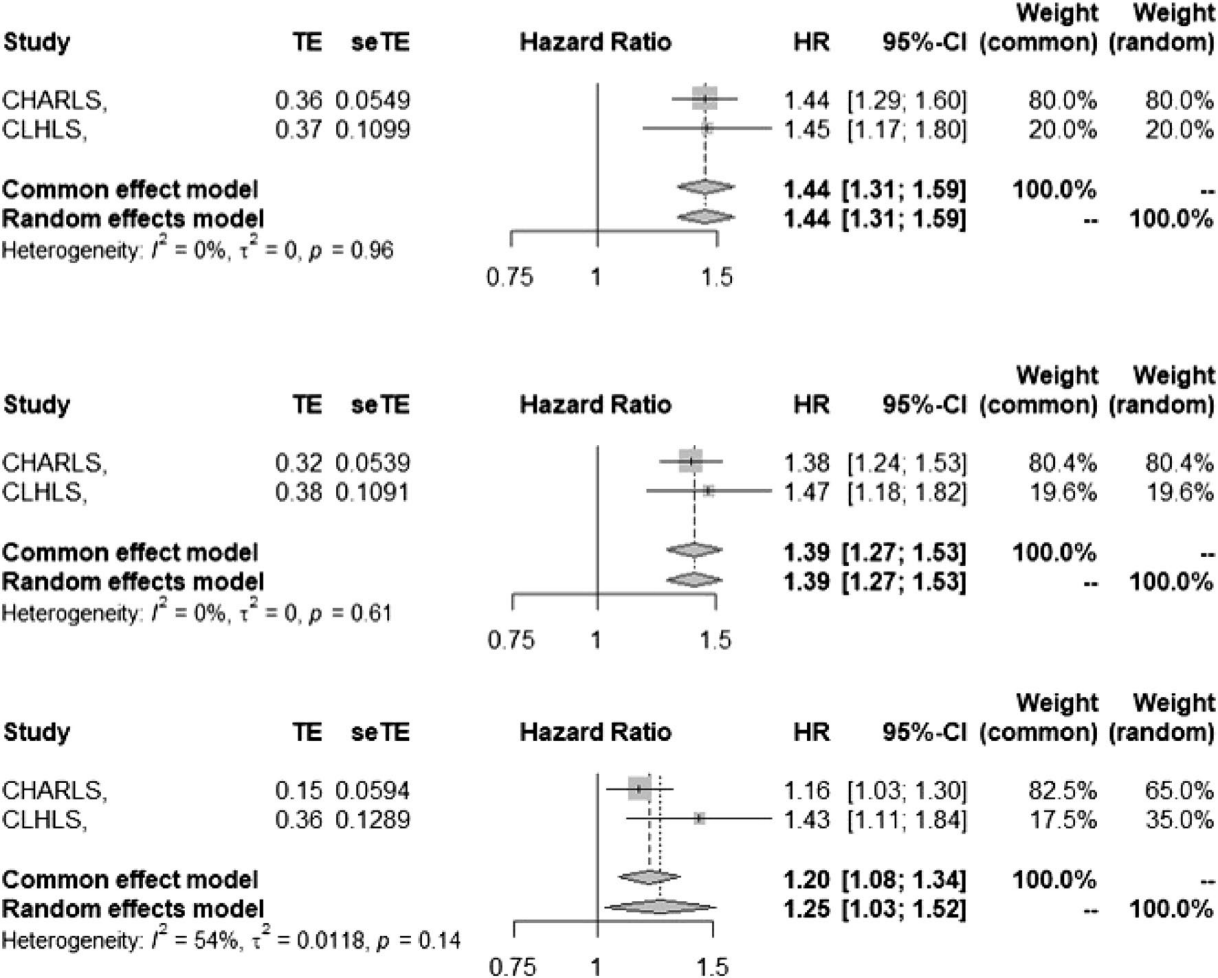
Combined HR values of multi-factor adjusted models.

The results obtained by cox regression analysis in both CHARLS and CLHLS cohort studies supported that solid fuel exposures caused higher risk of hearing loss compared with clean fuel, and HR (HR = 1.43) of CLHLS was higher than that of CHARLS (HR = 1.16). Although the outcome measures of the two cohorts were different, the exposure factors and covariates were the same, and the pooled HR value better reflects the effect of fuel on hearing loss and is more representative. Therefore, meta-analysis was used to merge HR values of three models. The heterogeneity of the results in the two cohorts was low (*P* > 0, I^2^ < 50%), so fixed models were used to merge the results. Unadjusted for confounders, the combined incidence of hearing loss exposed to solid fuel was 1.44 (1.31, 1.59) times higher than that of clean fuel; after adjustment for gender and age, the incidence of hearing loss exposed to solid fuel was 1.39 (1.27, 1.53) times that of clean fuel; further adjusting for confounding factors, the incidence of hearing loss exposed to solid fuel was 1.17 (1.07, 1.29) times higher than that of clean fuel. More details are shown in Fig. 5.

## Discussion

This study explored the relationship between solid fuel and hearing loss through two large cohorts (CHARLS and CLHLS) and got the same results that the adults using solid fuel for cooking had a higher risk of hearing loss compared with clean fuel users in China. The HR value (HR: 1.16, 95% CI 1.03–1.30, *P* < 0.05) from CHARLS cohort was similar to the other cohort study that included 8835 adults from CHARLS database (HR: 1.15, 95% CI 1.02–1.28)^11^. CLHLS cohort identified a higher cumulative prevalence rate and HR value (HR: 1.43, 95% CI 1.11–1.84, *P* < 0.01), which has not been previously investigated. Compared with CHARLS cohort, the participants of CLHLS cohort were mostly older than 65 years old (98.49%) while 18–65 years old participants in CHARLS accounted for 75.81%, this may explain the higher HR values in CLHLS cohort than in CHARLS cohort, indicating that the elderly is a high-risk group. Merging results showed that the incidence of hearing loss exposed to solid fuel was 1.17 (1.07, 1.29) times higher than that of clean fuel, which is more similar to the result of CHARLS. We could believe that solid fuel is a risk factor based on almost 12,000 adults’ data, and it will be severer in the elder. By the test efficiency, the result from the CHARLS is more credible than CLHLS, which means more sample of the elder needed. Many old people have the hearing loss before the start of the study, thus CLHLS’s sample size is smaller than CHARLS, I think more studies are needed to found the relationship in other countries and regions.

This study considered many risk factors of hearing loss that has been reported in epidemiological studies. One study analyzed Nurses’ Health Study II (1991–2013) found cigarette smoking was associated with higher risk of hearing loss, the MVRR (95% confidence interval) among past smokers with 20 + pack-years of smoking was 1.30 (1.09–1.55) and 1.21 (1.02–1.43) for current smokers^21^. Controversy over whether alcohol consumption is linked to hearing loss^22–24^. A study included 48,549 employees aged 20–64 years and free of hearing loss found that overweight (1.21, 1.08–1.36) and obesity (1.66, 1.33–2.08) would increase the risk of hearing loss^25^. The characteristics of people with hearing loss and people using solid fuel for cooking are similar in education level, residence and profession. After adjusting these risk factors and socioeconomic factors, the effect of solid fuel on hearing loss still exists independently. Compared with smoking and obesity, the influence of solid fuel is lower. A study reported higher systolic blood pressure was associated with hearing impairment at 1 kHz^26^ and a large cohort study of young and middle-aged men and women found that diabetic patients had a moderately increased risk of future hearing loss^27^. Some other research found fuel increased the risk of hypertension^28^ and diabetes^29^. In our current study, after adjusting hypertension and diabetes, there is no difference between the HR values of Model 3 and Model 4 both in CHARLS and CLHLS cohorts. This means that the mechanism by which solid fuels cause hearing loss has nothing to do with hypertension and diabetes, indicating that there may be other mechanisms that affect hearing loss. The significance of these variables adjusted in multivariate model was different in CHARLS cohort and CLHLS cohort, i.e., age significantly influenced the risk of hearing loss in CLHLS cohort, but those older, live in rural, with lower education level, with hypertension and not drinking adults had a higher risk of hearing loss in CHARLS cohort.

Air pollutants such as carbon monoxide (CO) and fine particulate matter (PM_2.5_) were produced during solid fuel combustion, which may be responsible for the hearing loss from solid fuel. A meta-analysis reported simultaneous exposure to noise and CO led to greater high-frequency noise-induced hearing loss (HFNIHL) than noise exposure alone (OR = 1.87, 95% CI 1.09–3.21)^30^. And another research found exposed in CO alone still increased risk of sudden sensorineural hearing loss^31^. Two studies from Taiwan^32^ and South Korea^33^ showed that high levels of PM_2.5_ would increase sudden sensorineural hearing loss, but a study from the United Kingdom^34^ showed a significant association between coarse particulate matter (PM_10_) and hearing impairment, whereas PM_2.5_ did not show similar associations. One of the underlying mechanisms may be oxidative stress. Studies have shown that CO can cause noise-induced hearing loss through oxidative stress^35^. In addition, CO-induced long-term chronic hypoxia can also cause irreversible damage to inner ear hair cells^36^. Olivetto et al.^37^ found that PM_2.5_ had the effects of pro-oxidative stress and inflammatory response, which affected ear inflammation and damages hair cells. People consciously avoid gas poisoning, but there is a lack of attention to the harmful substances produced by the combustion of solid fuels, which are often more difficult to avoid during cooking process.

Combined results of CHARLS and CLHLS studies provide further evidence that cooking using solid fuel is associated with an increased risk of hearing loss. It could be avoided through proactive policy action. People who use solid fuels are mainly located in rural areas with lower education levels. They are relatively weak in obtaining medical resources and are also in a disadvantaged position in terms of fuel use. Irrespective of exposure, subjects of low socio-economic status experience greater health effects of air pollution^38^. But the exposure of solid fuel, it increases health inequities. Sensory hearing loss is irreversible and there is no effective way to reverse the loss in neurological hearing loss^8^. Therefore, converting solid fuels to clean fuels can reduce the incidence of hearing loss and the burden of other diseases, meantime, promote health equity and impose higher economic effect.

The study has several limitations. Firstly, exposure time can’t be calculated, therefore, a dose–response relationship cannot be obtained. Secondly, we lack the data of cooking time, ventilation level, and general indoor air quality in investigated family, despite extensive adjustment for relevant variables in this study, residual confounders are still possible. Thirdly, this study doesn’t prove the age is the reason of difference between two cohort study. Besides, more studies are needed to merge to get a higher accurate result. However, this paper still has the advantage that it analyzed two large databases in China and combined study results are highly credible.

## Conclusion

Combined findings from CHARLS and CLHLS databases conclude that using solid fuels for cooking is associated with a higher risk of hearing loss than clean fuels in adults. Promoting the shift from solid fuels to clean fuels is of great significance for reducing hearing loss and promoting health equity.

## Data avalability

The access policy and procedures are available at www.ckbiobank.org.
